# The Tumor Suppressor PRDM5 Regulates Wnt Signaling at Early Stages of Zebrafish Development

**DOI:** 10.1371/journal.pone.0004273

**Published:** 2009-01-26

**Authors:** Natalia Meani, Federica Pezzimenti, Gianluca Deflorian, Marina Mione, Myriam Alcalay

**Affiliations:** 1 Department on Experimental Oncology, European Institute of Oncology, Milan, Italy; 2 Cogentech−Consortium for Genomic Technologies, Milan, Italy; 3 IFOM−FIRC Institute for Molecular Oncology Foundation, Milan, Italy; 4 Dipartimento di Medicina, Chirurgia ed Odontoiatria, Università degli Studi di Milano, Milan, Italy; Katholieke Universiteit Leuven, Belgium

## Abstract

*PRDM* genes are a family of transcriptional regulators that modulate cellular processes such as differentiation, cell growth and apoptosis. Some family members are involved in tissue or organ maturation, and are differentially expressed in specific phases of embryonic development. PRDM5 is a recently identified family member that functions as a transcriptional repressor and behaves as a putative tumor suppressor in different types of cancer. Using gene expression profiling, we found that transcriptional targets of PRDM5 in human U2OS cells include critical genes involved in developmental processes, and specifically in regulating wnt signaling. We therefore assessed PRDM5 function in vivo by performing loss-of-function and gain-of-function experiments in zebrafish embryos. Depletion of *prdm5* resulted in impairment of morphogenetic movements during gastrulation and increased the occurrence of the *masterblind* phenotype in *axin*+/− embryos, characterized by the loss of eyes and telencephalon. Overexpression of *PRDM5* mRNA had opposite effects on the development of anterior neural structures, and resulted in embryos with a shorter body axis due to posterior truncation, a bigger head and abnormal somites. In situ hybridization experiments aimed at analyzing the integrity of wnt pathways during gastrulation at the level of the prechordal plate revealed inhibition of non canonical PCP wnt signaling in embryos overexpressing *PRDM5,* and over-activation of wnt/β-catenin signaling in embryos lacking Prdm5. Our data demonstrate that PRDM5 regulates the expression of components of both canonical and non canonical wnt pathways and negatively modulates wnt signaling in vivo.

## Introduction

The human *PRDM* gene family consists of 17 known members characterized by the presence, generally at the N-terminus, of the PR domain, related to the SET domain functioning in chromatin-mediated transcriptional regulation [1], followed by a variable number of zinc finger repeats. Several studies suggest that PRDM family members are negative regulators of cell growth and tumorigenesis [2], [3], [4], [5], [6], [7], and their frequent inactivation in a broad spectrum of tumors largely supports this hypothesis [8], [9], [10], [11], [12], [13].

PRDM5 (or PFM2) is a recently characterized member of the PRDM family. Although its precise biological function remains to be elucidated, inactivation of *PRDM5* in different tumors suggests that it may behave as a tumor suppressor. It is, in fact, often silenced in cell lines derived from breast, ovarian and hepatic tumors [7] and has been identified as a target of epigenetic silencing in colorectal and gastric cancer [14]. PRDM5 may also have other disease-linked functions: two *PRDM5* sequence variants were recently found in a study of neutropenic patients that lacked mutations in genes associated to hereditary neutropenia, such as *ELA2* and *GFI1* [15].

PRDM5 acts as a sequence specific DNA-binding transcription factor, and its consensus DNA binding sequence has recently been described [15]. Its activity derives from the association with chromatin modifying enzymes, such as histone methyltransferase G9A and histone deacetylase 1 (HDAC1), which are recruited to its target promoters determining modifications in the methylation and acetylation status of chromatin [15].

We performed gene expression profiling after PRDM5 induction in U2OS cells, and found differential expression of genes involved in development and cell fate determination, such as components of wnt signaling pathways. An important role for other *PRDM* genes in embryonic development has been described through functional studies in different animal models, and is further supported by their specific and restricted pattern of expression during development [16], [17], [18]. The possibility that PRDM5, like other PRDM proteins, might regulate important developmental processes prompted us to investigate its role in zebrafish embryogenesis. Our results show that PRDM5 negatively modulates both the canonical wnt/β-catenin pathway and the non canonical planar cell polarity (PCP) wnt pathway in early stages of zebrafish development.

## Results

### Identification of genes regulated by PRDM5 in U2OS cells

**Figure 1:**
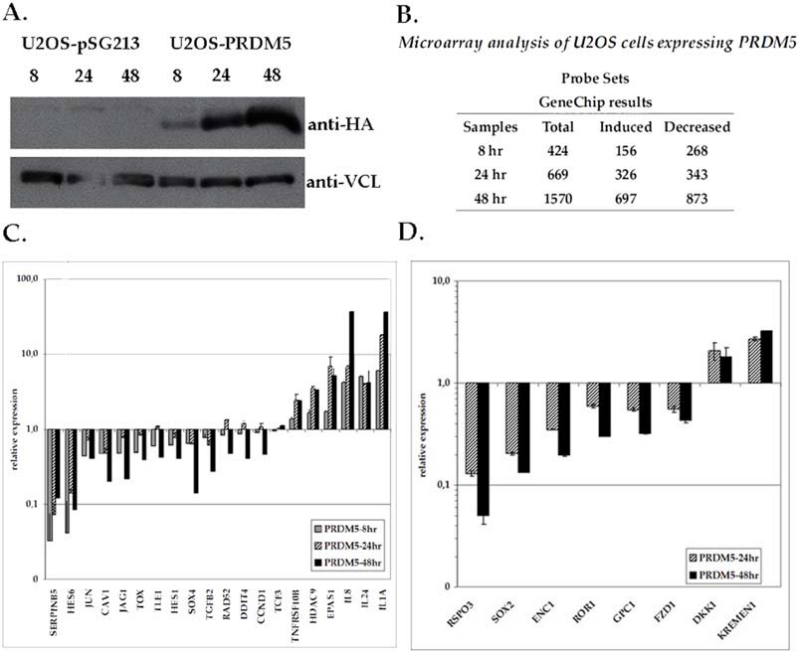
Gene expression profiling of a U2OS cell line overexpressing PRDM5. A. Western Blot analysis of PRDM5 protein expression in the U2OS-PRDM5 clone. PRDM5 protein level was analyzed 8, 24 and 48 hours after doxycycline treatment. U2OS cells bearing the empty vector (U2OS-pSG213) were used as control. HA-tagged PRDM5 was detected by immunoblot with an anti-HA antibody; anti-vinculin (anti-VCL) antibody was used for normalization of protein levels. B. Number of regulated Affymetrix probe sets identified by gene expression profiling at each time point. C–D. Expression level of PRDM5 target genes. Relative mRNA levels (logarithmic scale) of induced and repressed genes in U2OS cells after expression of PRDM5 for 8, 24 and 48 hours, as assayed by qPCR. A collection of PRDM5 target genes randomly selected (C) or of the wnt pathways (D) is shown.

We analyzed the gene expression profile of a U2OS cell line conditionally expressing HA-tagged PRDM5 (U2OS-PRDM5), where *PRDM5* is under the transcriptional control of a doxycycline-inducible promoter. Expression of PRDM5 protein is detectable after 8 hours of induction with 2 µg/ml doxycycline, and increases steadily up to 48 hours (Figure 1A), after which cells begin to undergo apoptosis (data not shown). U2OS cells containing the empty cloning vector (U2OS-pSG213) were used as controls for each condition.

Total RNA was extracted from U2OS-PRDM5 and U2OS-pSG213 cells after 8, 24 and 48 hours of doxycycline treatment. We performed four independent experiments for each time point and pooled the RNAs corresponding to individual experimental conditions. Biotinylated cRNA targets were synthesized from each RNA pool and hybridized to Affymetrix oligonucleotide chips (GeneChip HG-U133 Plus 2.0). Results were analyzed using GCOS and further elaborated with proprietary software, as described in Materials and Methods [19].

GeneChip probe sets regulated by PRDM5 at different time points are summarized in Figure 1B. Cross comparison of results identified 197 probe sets (59 induced and 139 repressed) that are concordantly regulated at all time points (Table S1). The number of target genes increases at later time points, probably due to the increasing amount of PRDM5 protein (Figure 1A).

To determine the reliability of microarray data, a random set of twenty genes was assayed by qPCR on an independent set of RNAs (Figure 1C). We found concordance between GeneChip prediction and expression level in the U2OS system for 19/20 genes (Figure 1C and Table S2).

### Functional classification of PRDM5 target genes

We next performed functional classification of regulated genes to gain insight on the cellular processes targeted by PRDM5 expression. First, we classified genes that are regulated at all time points by collecting information from Web-based sources (GOTM, gene ontology tree machine at http://bioinfo.vanderbilt.edu/gotm; SOURCE at http://source.stanford.edu/cgi-bin/source/sourceSearch; Gene and PubMed at http://www.ncbi.nlm.nih.gov/). The enriched functional categories included cell adhesion and development (Table S3).

Different genes encoding proteins involved in cell adhesion (*CNTN3, CNTNAP2, NINJ1*) [20], [21], components of extracellular matrix (*COL5A1, COL6A3, FBLN1, MFAP5*) [22], [23] or proteins involved in regulation of extra cellular matrix (ECM) production (*FOXF2*) [24] are down regulated, suggesting that PRDM5 negatively modulates cell-cell and cell-matrix adhesion. Expression of the gene encoding for metalloprotease ADAMTS6 is instead upregulated and may function by activating remodeling of ECM [25]. Cell-cell and cell-matrix interactions play an important role in the reception, coordination and modulation of external stimuli, resulting in the regulation of cell growth and survival.

Different genes expressed in the nervous system and/or involved in development are modulated by PRDM5 expression in U2OS cells. Of particular interest, we found changes in the expression levels of relevant components of wnt signaling pathways. We therefore searched more extensively for genes related to wnt signaling by analyzing the lists of PRDM5 target genes obtained at different time points, but not necessarily common to all. We looked for components of wnt signaling pathways as well as for wnt targets, exploiting data collected in the Wnt Homepage (http://www.stanford.edu/~rnusse/wntwindow.html), and found that 32 putative PRDM5 targets are included in one of these categories (Table S4). We confirmed microarray results through RT-PCR on a selected set of genes (Figure 1D and Table S2).

The wnt antagonists *KREMEN1* and *DKK1* [26] are upregulated upon PRDM5 expression, while two putative agonists, *RSPO3* and *SOX4* [27], [28], are downregulated, suggesting PRDM5 may antagonize wnt signaling. Consistently, a decrease in PRDM5 expression in U2OS cells by shRNA results in downregulation of *KREMEN1* and upregulation of *RSPO3* (data not shown). PRDM5 expression also results in decreased expression of wnt receptors *FZD1* and *FZD2* and of putative wnt targets, such as *CCND1*, *FST*, *FN1* and *ENC1*. Genes of the non canonical PCP wnt pathway, such as the co-receptor *ROR1*, are also regulated. These results suggest a role for PRDM5 in the regulation of both canonical and non canonical wnt signaling. Wnt signaling is involved in many aspects of embryonic development, such as morphogenetic movements, cell type specification and patterning. We therefore investigated the role of PRDM5 in zebrafish development.

### Expression pattern of *prdm5* during zebrafish development

**Figure 2:**
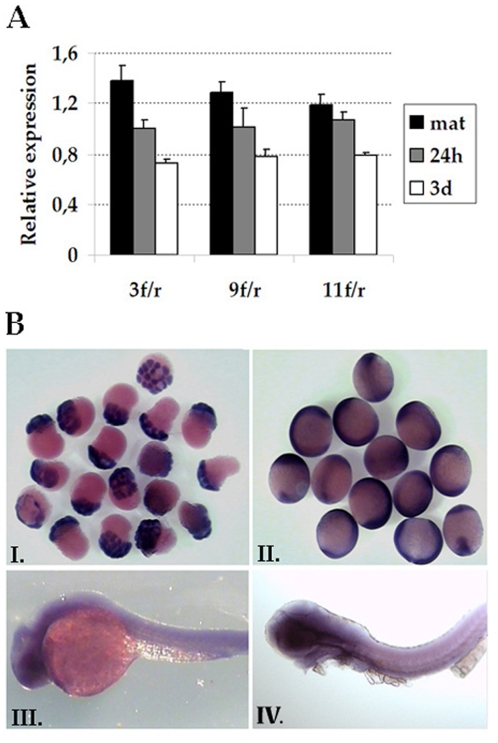
Expression of *prdm5* during Zebrafish development. A. Expression of *prdm5* was measured by qPCR using three pairs of primers (3f/r, 9f/r and 11f/r) that amplify different regions of the transcript. Relative expression levels at each developmental stage (2–8 cell stage = mat, 24 hours pf = 24h, 3days pf = 3d) were calculated with respect to the average expression levels in all samples. B. Whole mount *in situ* hybridization of 1 hpf (I), 9 hpf (II), 24 hpf (III) and 48 hpf (IV) zebrafish embryos stained with a cRNA dig-labeled *prdm5* probe. Ubiquitously high expression levels are detected in the first two stages; lower levels are present in 24 and 48 hpf.

Zebrafish Prdm5 protein is highly homologous to the human protein (Figure S1) with the PR domain at the N-terminus followed by a stretch of 16 zinc fingers (http://www.expasy.ch/prosite/). We first analyzed the expression of *prdm5* in developing zebrafish using RT-PCR and in situ hybridization in whole embryos 30 min to 48 hours post fertilization (hpf), and in sections of larvae 2 weeks post fertilization. A *prdm5* transcript corresponding to the full length coding region is present in the fertilized eggs and throughout development (Figure 2A). In situ hybridization on whole embryos ranging from 30 min to 48 hpf showed strong maternal expression (Figure 2B), followed by ubiquitous expression after the onset of zygotic transcription, with higher levels in the central nervous system (CNS) at 24 hpf and 48 hpf (Figure 2BIII-IV). In situ hybridization on sections of 2 week old fish showed that *prdm5* expression is restricted to specific tissues including intestinal mucosa, ventral spinal chord and ciliary zone (data not shown).

### Effects of *prdm5* knockdown and overexpression during embryogenesis

We next used both loss of function and gain of function approaches to study the role of Prdm5 during zebrafish development. We designed two morpholino oligonucleotides to obtain a depletion of Prdm5 protein: one targeting the region comprising the start codon (ATGmo), and one targeting the exon1/intron1 splice site (splice blocking or SBmo). It was not possible to assess the levels of residual protein through Western Blotting after depletion of Prdm5 using the ATGmo due to the lack of a suitable antibody. However, the phenotype was comparable to that caused by the SBmo, which achieved an almost complete block of splicing of the zygotic *prdm5* mRNA (Figure S2), predicted to result in a protein truncated at the level of amino acid 31 (at the N-terminus of the PR domain), and therefore predicted to be not-functional.

**Figure 3:**
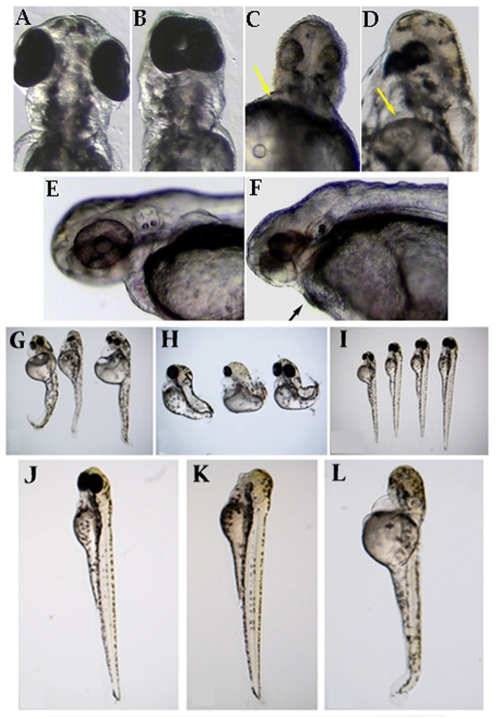
Phenotypic analysis of Prdm5 functions in zebrafish development. *A–F: Depletion of prdm5.* Cyclopia and other axial mesendodermal defects in *prdm5* morpholino injected zebrafish embryos. 48 hpf embryos either wild type (A,E) or injected (B: ATGmo; C: SBmo- 4ng; D: SBmo-8ng, F: Mix mo) are shown. Arrows in C, D and F point to mesendodermal defects. *G–I: Overexpression of hPRDM5 rescues the morpholino phenotype*. G: embryos injected with SBmo morpholino; H: Injections of 100 pg *hPRDM5* mRNA in zebrafish embryos results in a “dorsalized” phenotype. I: Co-injection of *prdm5* SBmo and *hPRDM5* mRNA results in normal embryos. 48 hpf embryos are shown. *J–L: Depletion of prdm5 enhances the mbl phenotype.* Effect of *prdm5* morpholino injection on the *mbl* phenotype. Injection of Mix mo in embryos derived from incrosses of *mbl* carriers. J) wt embryo; K) *mbl* homozygous mutants; L) *mbl* heterozygotes injected with Mix mo. All embryos analysed at 48 hpf.

The effects of the two morpholinos on zebrafish development were similar and consisted in the dose-dependent appearance of a cyclopic phenotype (Figure 3A–D). Doses lower than 2 ng yielded no visible phenotype, and doses higher than 10 ng resulted in strong underdevelopment of most embryos. There was a slight difference in the phenotypes induced by the two morpholinos. The ATGmo induced stronger cyclopia (Figure 3B), while the SBmo induced closer, smaller eyes and marked axial mesendodermal defects (jaw, heart and blood defects) (Figure 3C–D). The injection of a mixture of both morpholinos (Mix mo: 4ng ATGmo+4ng SBmo) gave a phenotype similar to the SBmo alone (Figure 3F). The differences in the effects of the two morpholinos possibly reflect different roles of maternal and zygotic Prdm5 in convergent extension (CE) movements of components of the mesendoderm.

Injection of mRNA encoding for human PRDM5 (*hPRDM5*) at doses ranging between 50 and 200 pg/embryo resulted in embryos with a shorter body axis due to posterior truncation, a bigger head and abnormal somites (Figure 3H), a phenotype somehow opposite to that induced by SBmo injections.

To verify if the defects observed in embryos injected with either *prdm5* morpholinos or *hPRDM5* were due to specific loss or gain of function of Prdm5, we co-injected SBmo (6 ng) and *hPRDM5* mRNA (150 pg) and assessed if the phenotype induced by either treatment was rescued. It is important to note that no sequence similarities exist between the morpholino and the mRNA. We found that 71/100 embryos injected with both SBmo and *hPRDM5* mRNA were normal (Figure 3I) as opposed to a very low number of normal embryos in clutches injected only with SBmo (8/110, Figure 3G), or only with *hPRDM5* mRNA (7/130, Figure 3H).

### Effect of *prdm5* depletion and *hPRDM5* overexpression in the *mbl* background

During gastrulation, the activation of canonical wnt/β-catenin signaling is required for regionalization of the anterior neural plate (ANP) that will generate telencephalic, eye field, diencephalic, and hypothalamic fates. A gradient of β-catenin signaling determines the identity of each domain, with high levels of signaling promoting more caudal neural identities. *Masterblind* (*mbl*) heterozygous zebrafish carry a null mutation in the *axin* gene, which encodes for an essential component of the β-catenin destruction complex. Homozygous mutants have a constitutively active canonical wnt pathway that results in posteriorization of the anterior brain and consequently in the absence of eyes and telencephalon [29]. To assess the role of Prdm5 in modulating wnt signaling, we assessed the effect of *prdm5* depletion or overexpression in embryos derived from incrosses of *mbl* (*axin*+/−) carriers.

Injections of 6 ng of a mixture of both *prdm5* morpholinos increased the number of embryos displaying the *mbl* phenotype from approximately 25% to >50%, thus indicating that a reduction of *prdm5* levels results in an increase of canonical wnt signaling, and overcomes the low levels of functional axin in heterozygotes (Table S5, Figure 3J–L). By contrast, injection of *hPRDM5* mRNA was not able to change the number of embryos with an *mbl* phenotype, but induced a shorter body and a bigger head in most embryos, regardless of the *mbl* phenotype. Therefore, in *axin+/−* embryos, the depletion of *prdm5* determines a significant increase in the frequency of the *mbl* phenotype, which is known to derive from an increase in canonical wnt signaling.

### *prdm5* modulates CE movements during embryogenesis

The cyclopic phenotype observed in *prdm5* depleted zebrafish embryos could be the consequence of a block of CE movements during gastrulation at the level of the prechordal plate [30]. To verify this hypothesis we performed triple in situ hybridization experiments using two different cocktails of cRNA probes: one consists of probes for *hgg1*, *ntl* and *dlx3*, and serves to identify CE defects due to impairment of the non-canonical wnt pathway [31]; the other contains probes for *rx3, pax2a* and *ntl,* and explores CE defects deriving from impairment of the canonical wnt pathway [32].

**Figure 4:**
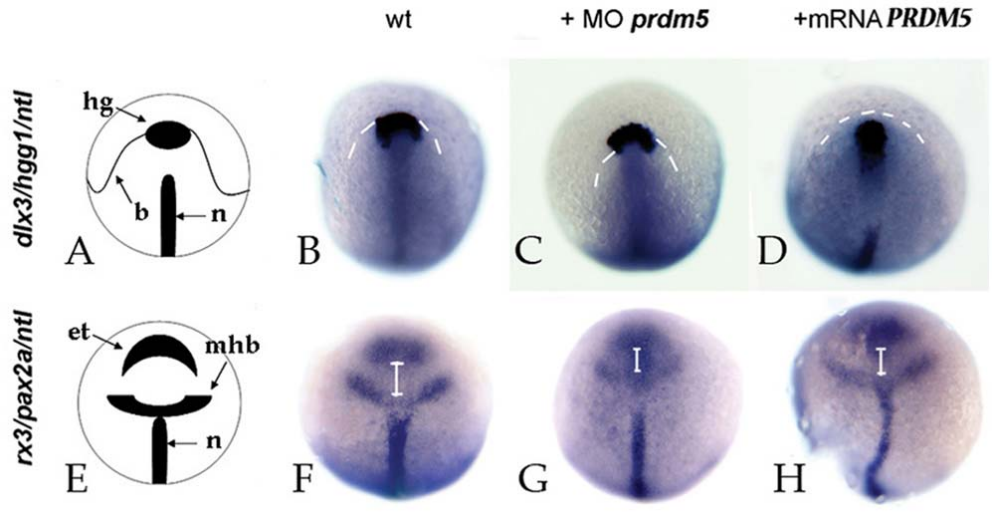
Analysis of CE movements at gastrulation. A, E = diagrams illustrating the staining pattern. B–D: Dorsal views of zebrafish embryos at 90% epiboly stage, stained for *hgg1* (h = rostral mesendoderm), *ntl* (n = notochord), *dlx3* (b = neural border). White dashed lines mark the *dlx3* signal, and correspond to the border between neural and non-neural ectoderm. F-H: dorsal views of zebrafish embryos at 90% epiboly stage, stained for *rx3* (e+t = eye field+telencephalon), *pax2a* (mhb = midhindbrain boundary) and *ntl* (n = notochord). The embryos were uninjected (B, F), injected with 6 ng SBmo (C, G) or 150 pg *hPRDM5* mRNA (D, H). White lines mark the distance between eye field and mid-hindbrain boundary.

In normal embryos, the most anterior structure is identified by the hatching gland (marked by *hgg1* expression) that always aligns with the border between neural and non-neural ectoderm (marked by *dlx3* expression) (Figure 4A–B). Zebrafish embryos, uninjected (Figure 4B), injected with 6 ng SBmo (Figure 4C) or with 150 pg *hPRDM5* mRNA (Figure 4D) were stained for *hgg1*, *ntl* and *glx3* at 90% epiboly stage. After overexpression of *hPRDM5*, the hatching gland is included in the neural border, an event that correlates with defects in migration of mesendodermal cells (Figure 4D). This behavior indicates inhibition of non canonical PCP wnt signaling, and suggests that Prdm5 negatively modulates this pathway.

The second cocktail stains three structures of the gastrulating embryo: the eye/telencephalon (*rx3*), the mid-hindbrain border (*pax2a*) and the notochord *(ntl*) (Figure 4E–F). Staining of zebrafish embryos injected with 6 ng SBmo (Figure 4G) or with 150 pg *hPRDM5* mRNA (Figure 4H) with this cocktail revealed that depletion of *prdm5* induces a reduction of the distance between eye/telencephalon and the mid-hindbrain border (Figure 4G), which is indicative of perturbation of morphogenetic movements as a consequence of over-activation of wnt/β-catenin signaling. Taken together our data suggest that *prdm5* inhibits wnt signaling (both canonical and non-canonical) in the anterior CNS and mesendoderm.

### *dkk1* expression is modulated by Prdm5 in early stages of zebrafish development

Since *DKK1* is upregulated by PRDM5 in U2OS cells, and considering that injection of *hPRDM5* mRNA in zebrafish embryos results in a phenotype similar to that described after Dkk1 overexpression [33], we assessed *dkk* expression following *prdm5* morpholino or *hPRDM5* mRNA injections by *in situ* hybridization.

**Figure 5:**
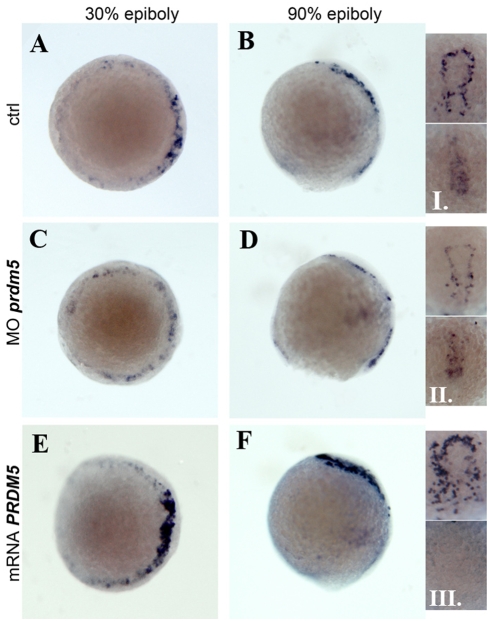
Prdm5 regulates *dkk1* expression in early zebrafish embryos. Expression of *dkk1* at 30% epiboly (3 hpf) A, C, E and at 90% epiboly (6 hpf) B, D, F and insets I–III. A, C, E and upper panels of insets I–III are dorsal views (presumptive shield region to the right); B, D, F are lateral views (dorsal to the top). Lower panels of insets I–III show the tail region. A, B and inset I correspond to wt embryos, C, D and inset II to embryos depleted for *prdm5* by injection of Mix mo and E, F and inset III to embryos injected with *hPRDM5* mRNA.

Expression of *dkk1* in the two experimental conditions was evaluated at 30% and 90% epiboly, corresponding to 3 and 6 hpf, respectively. At both stages, injection of *hPRDM5* mRNA induced a marked increase in the level of expression of *dkk1* and in the number of presumptive mesodermal cells expressing it (Figure 5A–B, E–F). By contrast, injection of a mixture of both morpholinos (Mix mo: 4ng ATGmo+4ng SBmo) resulted in a reduction of *dkk1* expression. (Figure 5C–D). At 90% epiboly, in the embryos injected with *hPRDM5* mRNA the increase of *dkk1* expression was predominantly in the anterior mesendodermal region (Figure 5, upper panels of insets I–III), whereas *dkk1* expression in the presumptive tail mesoderm was strongly reduced (Figure 5, lower panels of insets I–III). The increase of *dkk1* in the head region, and the reduction of *dkk1* in the tail region of *hPRDM5* mRNA injected embryos result in the “rostralized” phenotype observed at 24–48 hpf. Our results suggest that regulation of *dkk* expression may participate to Prdm5-dependent inhibition of canonical wnt/β-catenin signaling.

## Discussion

We investigated PRDM5 function by identifying its target genes in U2OS cells, and we found that the two main functional categories of genes regulated by PRDM5 are molecules involved in cell-cell interactions and components of developmental signaling pathways. Cell-cell interactions are relevant in modulating the response to external stimuli, and modifications of membrane and/or matrix components may activate signaling events that change cell fate [34].

However, even more striking was the regulation of genes encoding for components of developmental signaling pathways. In particular, overexpression of PRDM5 in U2OS cells affects the transcription of both canonical and non canonical wnt pathway components. Wnt signaling is involved in many aspects of embryonic development, such as morphogenetic movements, cell type specification and patterning. Other *PRDM* genes exert important functions in development, as largely demonstrated by diverse studies in animal models [16], [17], [18]. We therefore investigated the role of PRDM5 in zebrafish development.

We found that downregulation of Prdm5 expression in zebrafish embryos affects CE movements leading to cyclopia or small eyes and axial mesodermal defects. On the contrary, *hPRDM5* mRNA overexpression results in a big head and short body axis, similar to the phenotype observed after overexpression of the wnt antagonist, Dkk1. In fact, Dkk1 behaves as “head inducer” antagonizing the posteriorizing effect of the canonical wnt pathway on anterior neural structures [33]. Coherently, Dkk1 is a target of upregulation by PRDM5 in U2OS cells.

The interplay between wnt/β-catenin and PCP signaling is fundamental for correct specification of anterior neural structures. While wnt/β-catenin cascade has a posteriorizing effect on anterior neural ectoderm and suppresses more rostral forebrain fates, PCP signaling antagonizes wnt/β-catenin, allowing specification of eye field and telencephalon [32]. Dkk1 acts by interfering with wnt/β-catenin signaling and favoring PCP signaling, thereby functioning to bridge the two pathways [33]. How Dkk1 favours PCP signalling is not clear, but it may cooperate with the glyplican 4/6 homologue Knypek to activate the signalling cascade. Glyplicans are cell-surface heparan sulfate proteoglycans that exert important functions in development by controlling different signalling pathways [35], such as the wnt pathway. Indeed, GPC3 selectively improves non-canonical wnt/PCP signalling, while inhibiting canonical wnt signalling in mouse models [36].

In zebrafish, Prdm5 acts by interfering with both signaling cascades, introducing an additional level of modulation to the complex scenario of wnt pathway regulation. We can speculate that Prdm5-dependent inhibition of wnt/β-catenin signaling is at least in part correlated to the increased expression of Dkk1. How Prdm5 inhibits non-canonical PCP signaling is, instead, unclear. Interestingly, we found that GPC1 is downregulated upon PRDM5 expression in U2OS cells. Although a role for GPC1 in the regulation of wnt signalling has not been reported so far, it is possible that PRDM5 may down modulate non-canonical wnt/PCP signalling through downregulation of glypicans, counteracting the positive effect of Dkk1 on this pathway.

The definition of PRDM5 as candidate tumor suppressor derives from its frequent inactivation in human cancers and from its ability to impair cell growth and enhance apoptosis. Constitutive activation of wnt signaling is a common event in human cancers, and alterations of specific pathway components have been implicated in diverse tumor types [37]. Our results, derived from gene expression data and functional studies in zebrafish development, suggest that PRDM5 may exert its tumor suppressor functions through negative modulation of wnt signaling. Inactivation of *PRDM5* through gene deletions, epigenetic silencing or point mutations may therefore represent a novel mechanism of constitutive activation of wnt signaling in human tumors.

## Materials and Methods

### Cell lines

The U2OS-PRDM5 cell line was generated by stable transfection of U2OS cells with human *HA-PRDM5* cDNA cloned in the pSG213 doxycyclin inducible vector; single clones were selected with 1,5 µg/ml puromycin for three days and then expanded in tetracyclin free medium. A bulk population of U2OS cells transfected with the empty vector (U2OS-pSG213) was used as a control. U2OS-PRDM5 and U2OS-pSG213 cells were maintained in DMEM supplemented with 100 µg/ml streptomycin, 100 µg/ml penicillin, 2 mM glutamine, 10% tetracyclin free serum and 1,5 µg/ml puromycin at 37°C in a humidified atmosphere containing 5% CO_2._ Doxycycline treatments were performed by adding 2 µg/ml doxycycline to the culture medium for 8, 24 or 48 hours.

### Constructs

Human *PRDM5* CDS was amplified by PCR using specific primers modified at the 5′ end with the sequence recognized by BamH1 restriction enzyme (B-PR5f1: 5′-(GC)GGATCCCTGGGCATGTACGTGCCGGA-3′; B-PR5r1: 5′-(GC)GGATCCTTAGCTGTCAGCTACACCAT-3′) and cloned into the BamH1 site of the pCDNA3-HA vector (Invitrogen). The HA-tagged *PRDM5* fragment was purified after digestion with SpeI and XhoI restriction enzymes cloned into the pSG213 vector. Human *PRDM5* CDS used for RNA synthesis was cloned in the BamH1 site of pCS2+ vector. A partial zebrafish *prdm5* CDS was cloned by PCR from 25 ng of cDNA derived from 3 day post fertilization embryos using the following primers: 5′-ACATGGATGATCAGCCTGGACT-3′ (DRPR5-1F) and 5′-TGTGTGTGCGCATGTGTTCGT-3′ (DRPR5-12R) and cloned into pCR2.1-TOPO vector.

### Affymetrix GeneChip hybridization

U2OS-PRDM5 and U2OS-pSG213 cells were treated with 2 µg/ml doxycycline for 8, 24 and 48 hours. Four independent inductions were performed and total RNA extracted using the RNeasy mini kit (Qiagen) to generate RNA pools for each condition. Biotin-labeled cRNA targets were obtained from 5 µg of each RNA pool using Affymetrix custom kit. GeneChip hybridization, washing, staining and scanning was performed according to Affymetrix (Santa Clara, California, USA). Two copies of the HG-U133 Plus 2.0 were hybridized with each target. Results were analyzed using GCOS (Affymetrix) and further elaborated through replica analysis and statistical methods using the GenePicker software [19]. Raw data can be accessed from the GEO repository (http://www.ncbi.nlm.nih.gov/geo/)with Data Set accession number GSE10580.

### Real-Time PCR

Validation of Affymetrix data was performed using the following collection of Taqman assays-on-demand by Applied Biosystems: CAV1: Hs00184697_m1; CCND1: Hs00277039_m1; DDIT4: Hs00430304_g1; DKK1: Hs00183740_m1; ENC-1: Hs00171580_m1; EPAS1: Hs00181674_m1; FZD1: Hs00268943_s1; GPC1: Hs00892476_m1; HDAC9: Hs00206843_m1; HES1: Hs00172878_m1; HES6: Hs00610927_g1; IL1A: Hs00174092_m1; IL24: Hs00169533_m1; IL8: Hs00174103_m1; JAG1: Hs00164982_m1; JUN: Hs00357891_s1; KREMEN1: Hs00230750_m1; RAD52: Hs00172536_m1; ROR1: Hs00178178_m1; RSPO3: Hs00262176; SERPINB5: Hs00184728_m1; SOX2: Hs00602736_s1; SOX4: Hs00268388_s1; TCF3: Hs00413032_m1; TGFB2: Hs00234244_m1; TLE1: Hs00270768_m1; TNFRSF10B: Hs00187196_m1; TOX: Hs00207075_m1.

Zebrafish *prdm5* expression was quantified using the SYBR green chemistry (Applied Biosystems) and three specific pairs of oligos: primer pair 3: forward 5′-GACGGGATGGGACTGTACACTA-3′; reverse 5′-CCATGCTTTCATCCAGATCACC-3′; primer pair 9: forward 5′-AGCTCACAGTCCAGTTTCCTGCA-3′; reverse 5′-TGCGAGCACCACCTATATGGAT-3′; primer pair 11: forward 5′-ATTCAGAGGAGAGGCCTTTCCA-3′; reverse 5′-TGGCGTCACAATGGTCACACTT-3′; β-actin1: forward 5′-CCACCATGAAGATCAAGATC-3′; reverse 5′-ACATCTGCTGGAAGGTGGA-3′).

Thermal cycling parameters were: 1 cycle at 50°C for 2 min, followed by 40 cycles in which the temperature ramp from 95°C to 60°C in 1 min. Each sample was run in triplicate. The mean value of replicates for each sample was calculated, expressed as cycle threshold and, for each sample, the C_T_ value of the endogenous control (18S or β-actin1) was subtracted to the C_T_ value of the target gene (ΔCt) to obtain comparable values. The relative amount of gene expression was calculated as the difference (ΔΔCt) between ΔCt of the test sample and of the control sample. Finally, the relative expression is expressed as 2^−ΔΔCt^.

### Ethics statement

Fish were maintained/raised according to EU regulations on laboratory animals.

### Strains and maintenance

Zebrafish strains were maintained and bred according to standard procedures. Tüebingen wild-type lines and axint^m213^ (previously known as *masterblind* (*mbl*)) mutant lines were used.

### In situ hybridization

Whole mount and section in situ hybridization was performed as previously described [38]. The *dkk1* probe was synthesized as previously described (Hashimoto et al., 2000). *prdm5* mRNA probe was synthesized as follows: CR2.1-TOPO-prdm5 vector was linearized with BamH1 or HindIII restriction enzymes, in vitro transcribed with T7 RNA polymerase, and the mRNA probe was subsequently labeled with DIG-RNA labeling mix (Roche). Anti-Digoxigenin-AP antibody (Roche) was then used to detect the hybridization signals.

### Antisense morpholino oligonucleotides and RNA injection

Antisense morpholino oligonucleotides were designed (GeneTools, LLC) against the first ATG of *prdm5* (ATGmo) or against the exon1-intron1 splice site, specifically targeting zygotic *prdm5* (Splicing Block MO, SBmo). The sequences of ATGmo and SBmo are 5′-TCCGGCACGTACATACCCAACATCC-3′ and 5′-TTATAGGCACGAACCTTCTTGACTG-3′, respectively.

Human *PRDM5* mRNA was synthesized from pCS2+-PRDM5 vector, previously linearized with NotI restriction enzyme. 10 µg of linearized vector were in vitro transcribed with SP6 using the mMessage Machine kit (Ambion). Embryos were microinjected at 1-cell stage using 50 to 200 pg of synthetic human *PRDM5* capped mRNA or 2 to 8 ng of ATGmo or SBmo.

## Supporting Information

Figure S1 PRDM5 protein is well conserved during evolution. ClustalX alignment of human (NP_061169), mouse (NP_081823) and Zebrafish (NP_001002301) PRDM5 proteins shows it is well conserved among human, mouse and zebrafish, with the exception of human exon 6 coding region (red box), which is poorly conserved between human and zebrafish and absent in the mouse homolog. The PR domain is located between the two arrows.
(2.89 MB TIF)

Figure S2 Efficiency of prdm5 knockdown assayed by the RT-PCR in SB mo injected embryos. Relative expression of prdm5 was measured by RT-PCR using three pairs of primers (see Materials and methods); relative expression at each developmental stage (2–8 cell stage = maternal, 24 hours pf = 24hpf, 3days pf = 3dpf) was calculated with respect to the not injected embryo (C) at the same stage.
(0.13 MB TIF)

Table S1 GeneChip probe sets regulated by PRDM5 expression in U2OS cells.
(0.42 MB DOC)

Table S2 Validation of microarray results by qPCR. GeneChip predictions are shown in the first three columns. Fold changes (FC) predicted by Affymetrix and by qPCR are shown.
(0.09 MB DOC)

Table S3 Functional classification of genes regulated by PRDM5 in U2OS cells.
(0.16 MB DOC)

Table S4 PRDM5 target genes of the wnt pathway.
(0.08 MB DOC)

Table S5 prdm5 depletion enhances masterblind phenotype in axin+/− zebrafish embryos. Three independent experiments of rescue of the mbl phenotype are shown. Control: not injected embryos; Mix mo: embryos injected with ATG and SB mo; mRNA: embryos injected with hPRDM5 mRNA. The expected percentage of normal or mbl embryos is shown (EXPECTED); the number (and percentage) of embryos obtained in each experiment and the corresponding phenotype is reported (EXP1, EXP2, EXP3, PHENOTYPE).
(0.04 MB DOC)
