# Supplementary figures and images for: The Tumor Suppressor PRDM5 Regulates Wnt Signaling at Early Stages of Zebrafish Development
Source: PLoS One. 2009 Jan 26;4(1):e4273. doi: 10.1371/journal.pone.0004273 (PMC2627919; doi:10.1371/journal.pone.0004273)

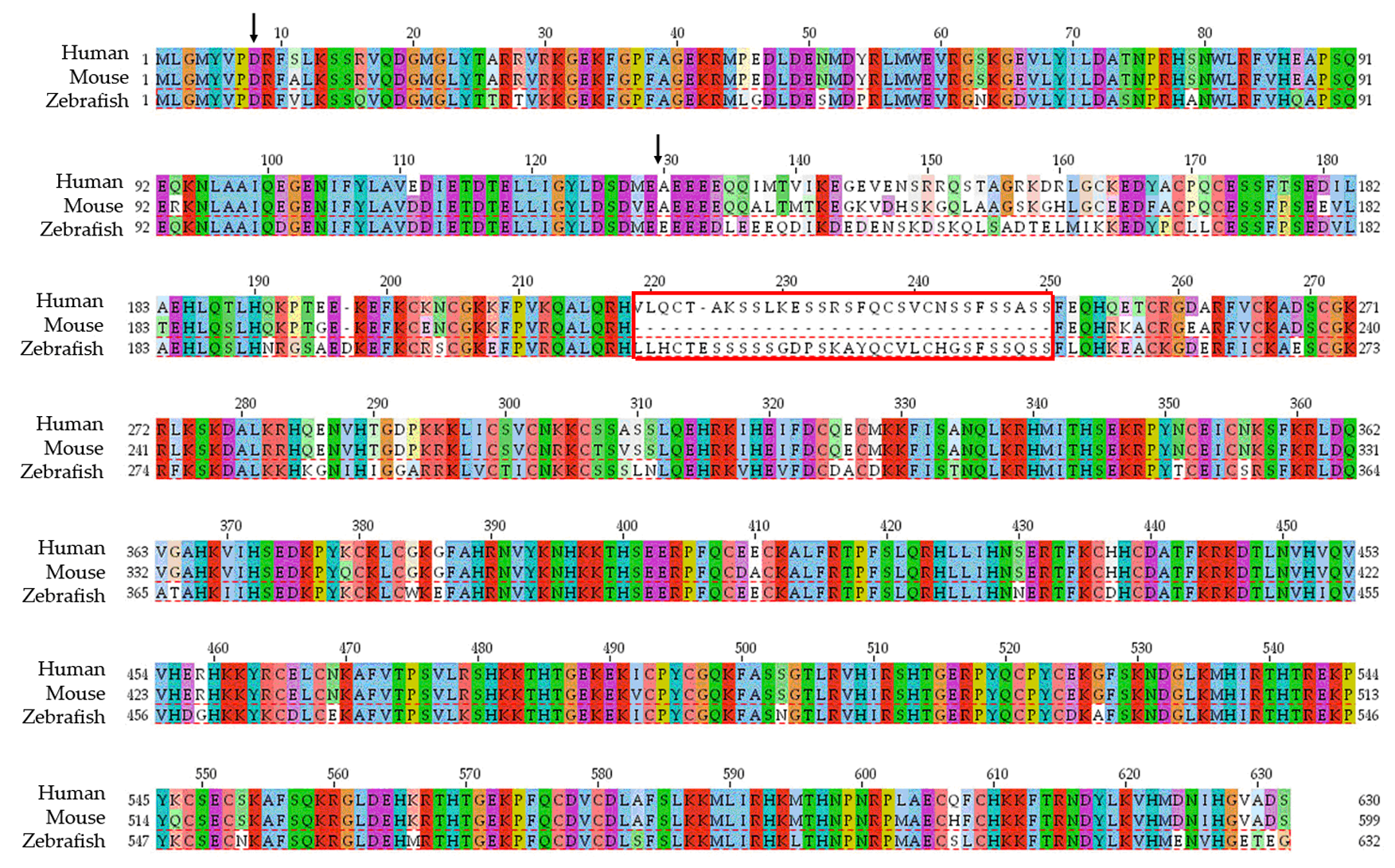

Supplement: Figure S1 — PRDM5 protein is well conserved during evolution. ClustalX alignment of human (NP_061169), mouse (NP_081823) and Zebrafish (NP_001002301) PRDM5 proteins shows it is well conserved among human, mouse and zebrafish, with the exception of human exon 6 coding region (red box), which is poorly conserved between human and zebrafish and absent in the mouse homolog. The PR domain is located between the two arrows. (2.89 MB TIF) [file pone.0004273.s001.tif]

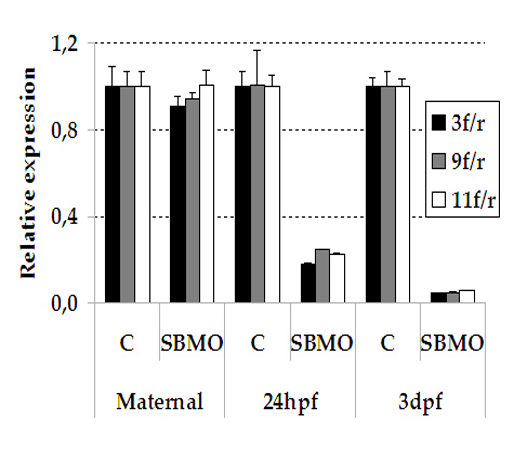

Supplement: Figure S2 — Efficiency of prdm5 knockdown assayed by the RT-PCR in SB mo injected embryos. Relative expression of prdm5 was measured by RT-PCR using three pairs of primers (see Materials and methods); relative expression at each developmental stage (2–8 cell stage = maternal, 24 hours pf = 24hpf, 3days pf = 3dpf) was calculated with respect to the not injected embryo (C) at the same stage. (0.13 MB TIF) [file pone.0004273.s002.tif]
