# Supplementary material for: The Tumor Suppressor PRDM5 Regulates Wnt Signaling at Early Stages of Zebrafish Development
Source: PLoS One. 2009 Jan 26;4(1):e4273. doi: 10.1371/journal.pone.0004273 (PMC2627919; doi:10.1371/journal.pone.0004273)
Supplement: Table S1 — GeneChip probe sets regulated by PRDM5 expression in U2OS cells. (0.42 MB DOC) [file pone.0004273.s003.doc]

**Table S1.** GeneChip probe sets regulated by PRDM5 expression in U2OS cells

| **Probeset** | **Symbol** | **Reg.** | **8hr** | **24hr** | **48hr** | **Accession** | **Name** |
| --- | --- | --- | --- | --- | --- | --- | --- |
| 1556134_a_at | B3GNT5 | **up** | 17.19 | 2.37 | 3.97 | BC013229 | UDP-GlcNAc:βGal beta-1,3-N-acetylglucosaminyltransferase 5 |
| 213782_s_at | MYOZ2 | **up** | 6.75 | 5.22 | 5.2 | BF939176 | Myozenin 2 |
| 211796_s_at | PRSS1 | **up** | 6.55 | 3.62 | 2.36 | AF043179 | Protease, serine, 1 (trypsin 1) |
| 205563_at | KISS1 | **up** | 6.19 | 4.88 | 4.3 | NM_002256 | KiSS-1 metastasis-suppressor |
| 207148_x_at | MYOZ2 | **up** | 6.12 | 5.39 | 5.97 | NM_016599 | Myozenin 2 |
| 213568_at | OSR2 | **up** | 5.47 | 4.26 | 3.17 | AI811298 | Odd-skipped related 2 (Drosophila) |
| 1557544_at | C10orf80 | **up** | 5.28 | 3.25 | 2.83 | BC036225 | Chromosome 10 open reading frame 80 |
| 209839_at | DNM3 | **up** | 5.2 | 5.86 | 6.28 | AL136712 | Dynamin 3 |
| 1558502_s_at | DNM3 | **up** | 4.98 | 3.62 | 4.4 | AI631915 | Dynamin 3 |
| 227705_at | TCEAL7 | **up** | 4.84 | 9.05 | 6.62 | BF591534 | Transcription elongation factor A (SII)-like 7 |
| 1558501_at | DNM3 | **up** | 4.7 | 4 | 4.22 | AI631915 | Dynamin 3 |
| 202957_at | HCLS1 | **up** | 3.89 | 4.77 | 3.11 | NM_005335 | Hematopoietic cell-specific Lyn substrate 1 |
| 222945_x_at | OLAH | **up** | 3.88 | 3.51 | 6.55 | AI125696 | Oleoyl-ACP hydrolase |
| 233126_s_at | OLAH | **up** | 3.74 | 5.13 | 6.5 | AK001844 | Oleoyl-ACP hydrolase |
| 226612_at | FLJ25076 | **up** | 3.67 | 3.94 | 2.26 | H17038 | Similar to CG4502-PA |
| 208180_s_at | HIST1H4H | **up** | 3.67 | 2.93 | 2.64 | NM_003543 | Histone cluster 1, H4h |
| 231947_at | MYCT1 | **up** | 3.6 | 3.13 | 7.6 | AI242583 | Myc target 1 |
| 218280_x_at | HIST2H2AA3 | **up** | 3.54 | 2.83 | 2.73 | NM_003516 | Histone cluster 2, H2aa3 |
| 222963_s_at | IL1RAPL1 | **up** | 3.5 | 2.76 | 6.19 | AF284435 | Interleukin 1 receptor accessory protein-like 1 |
| 203889_at | SCG5 | **up** | 3.5 | 2.11 | 2.55 | NM_003020 | Secretogranin V (7B2 protein) |
| 232035_at | HIST1H4H | **up** | 3.48 | 2.64 | 2.88 | BE740761 | Histone cluster 1, H4h |
| 221572_s_at | SLC26A6 | **up** | 3.32 | 4.01 | 4.09 | AF288410 | Solute carrier family 26, member 6 |
| 228646_at | PPP1R1C | **up** | 3.14 | 3.09 | 4.49 | AI806944 | Protein phosphatase 1, regulatory (inhibitor) subunit 1C |
| 237411_at | ADAMTS6 | **up** | 3.09 | 2.46 | 3.48 | N71063 | ADAM metallopeptidase with thrombospondin type 1 motif, 6 |
| 211371_at |  | **up** | 3 | 2.27 | 2.85 | U71088 |  |
| 220663_at | IL1RAPL1 | **up** | 2.99 | 2.98 | 5.04 | NM_014271 | Interleukin 1 receptor accessory protein-like 1 |
| 204852_s_at | PTPN7 | **up** | 2.95 | 2.37 | 4.56 | NM_002832 | Protein tyrosine phosphatase, non-receptor type 7 |
| 214455_at | HIST1H2BG | **up** | 2.94 | 2.26 | 2.38 | NM_003526 | Histone cluster 1, H2bg |
| 214290_s_at | HIST2H2AA3 | **up** | 2.93 | 2.3 | 2.3 | AI313324 | Histone cluster 2, H2aa3 |
| 227166_at | DNAJC18 | **up** | 2.89 | 6.07 | 5.68 | AI017750 | DnaJ (Hsp40) homolog, subfamily C, member 18 |
| 202859_x_at | IL8 | **up** | 2.88 | 2.73 | 13.25 | NM_000584 | Interleukin 8 |
| 211506_s_at |  | **up** | 2.88 | 2.6 | 19.7 | AF043337 |  |
| 232523_at | MEGF10 | **up** | 2.88 | 2.32 | 2.18 | AU144892 | Multiple EGF-like-domains 10 |
| 222803_at | PRTFDC1 | **up** | 2.78 | 2.46 | 2 | AI871620 | Phosphoribosyl transferase domain containing 1 |
| 235818_at | VSTM1 | **up** | 2.76 | 2.78 | 2.69 | AI498747 | V-set and transmembrane domain containing 1 |
| 203828_s_at | IL32 | **up** | 2.73 | 2.11 | 2.51 | NM_004221 | Interleukin 32 |
| 1555462_at | PPP1R1C | **up** | 2.72 | 2.19 | 17.41 | AF494535 | Protein phosphatase 1, regulatory (inhibitor) subunit 1C |
| 206120_at | CD33 | **up** | 2.65 | 4.77 | 4.47 | NM_001772 | CD33 molecule |
| 221911_at | ETV1 | **up** | 2.6 | 3.51 | 3.56 | BE881590 | Ets variant gene 1 |
| 206084_at | PTPRR | **up** | 2.6 | 2.26 | 2.42 | NM_002849 | Protein tyrosine phosphatase, receptor type, R |
| 210675_s_at | PTPRR | **up** | 2.55 | 2.22 | 2.73 | U77917 | Protein tyrosine phosphatase, receptor type, R |
| 235350_at | C4orf19 | **up** | 2.46 | 2.15 | 2.08 | AI935586 | Chromosome 4 open reading frame 19 |
| 205476_at | CCL20 | **up** | 2.44 | 3.2 | 10.03 | NM_004591 | Chemokine (C-C motif) ligand 20 |
| 208181_at | HIST1H4H | **up** | 2.39 | 2.41 | 2.3 | NM_003543 | Histone cluster 1, H4h |
| 1568933_at |  | **up** | 2.39 | 2.26 | 2.23 | CA424969 | CDNA clone IMAGE:5274919 |
| 236517_at | MEGF10 | **up** | 2.38 | 2.56 | 2.56 | AI968440 | Multiple EGF-like-domains 10 |
| 222838_at |  | **up** | 2.34 | 2.35 | 2.84 | AL121985 |  |
| 204602_at | DKK1 | **up** | 2.33 | 2.66 | 7.23 | NM_012242 | Dickkopf homolog 1 (Xenopus laevis) |
| 230746_s_at | STC1 | **up** | 2.31 | 2.89 | 2.94 | AW003173 | Stanniocalcin 1 |
| 226213_at | ERBB3 | **up** | 2.26 | 2.89 | 2.42 | AV681807 | V-erb-b2 erythroblastic leukemia viral oncogene homolog 3 |
| 219450_at | C4orf19 | **up** | 2.26 | 2.43 | 2.46 | NM_018302 | Chromosome 4 open reading frame 19 |
| 1570351_at |  | **up** | 2.26 | 1.97 | 2.42 | BC020916 |  |
| 1569886_a_at |  | **up** | 2.23 | 2.64 | 1.94 | BC040605 |  |
| 227048_at | LAMA1 | **up** | 2.22 | 2.04 | 2.42 | AI990816 | Laminin, alpha 1 |
| 228425_at | PAX8 | **up** | 2.18 | 2.16 | 4.49 | BF056746 | Paired box gene 8 |
| 202283_at | SERPINF1 | **up** | 2.11 | 2.48 | 2.74 | NM_002615 | Serpin peptidase inhibitor, clade F (alpha-2 antiplasmin, pigment epithelium derived factor), member 1 |
| 206549_at | INSL4 | **up** | 2.07 | 2.04 | 2.57 | NM_002195 | Insulin-like 4 (placenta) |
| 204064_at | THOC1 | **up** | 2.07 | 2 | 2.07 | NM_005131 | THO complex 1 |
| 219159_s_at | SLAMF7 | **up** | 2.04 | 1.93 | 2.56 | NM_021181 | SLAM family member 7 |
| 216048_s_at | RHOBTB3 | **down** | -1.97 | -2.07 | -2.07 | AK023621 | Rho-related BTB domain containing 3 |
| 204849_at | TCFL5 | **down** | -1.97 | -2.11 | -4 | NM_006602 | Transcription factor-like 5 (basic helix-loop-helix) |
| 227690_at | GABRB3 | **down** | -1.97 | -2.39 | -3.33 | BE502537 | Gamma-aminobutyric acid (GABA) A receptor, beta 3 |
| 212488_at | COL5A1 | **down** | -2 | -2.07 | -3.74 | N30339 | Collagen, type V, alpha 1 |
| 201341_at | ENC1 | **down** | -2 | -2.11 | -2.55 | NM_003633 | Ectodermal-neural cortex (with BTB-like domain) |
| 227646_at |  | **down** | -2 | -2.15 | -3.46 | BG435302 |  |
| 237737_at |  | **down** | -2 | -2.38 | -2.38 | AI359676 | Full length insert cDNA clone ZA88B06 |
| 229724_at | GABRB3 | **down** | -2 | -2.55 | -3.19 | AI693153 | Gamma-aminobutyric acid (GABA) A receptor, beta 3 |
| 205157_s_at | KRT17 | **down** | -2 | -2.6 | -4.14 | NM_000422 | Keratin 17 |
| 219872_at | C4orf18 | **down** | -2 | -2.65 | -3.62 | NM_016613 | Chromosome 4 open reading frame 18 |
| 203065_s_at | CAV1 | **down** | -2 | -2.69 | -3.48 | NM_001753 | Caveolin 1, caveolae protein, 22kDa |
| 1558643_s_at | EDIL3 | **down** | -2 | -2.73 | -3.61 | AA297258 | EGF-like repeats and discoidin I-like domains 3 |
| 204457_s_at | GAS1 | **down** | -2 | -3.32 | -5.98 | NM_002048 | Growth arrest-specific 1 |
| 214591_at |  | **down** | -2.02 | -3.52 | -3.15 | BF215673 |  |
| 203045_at | NINJ1 | **down** | -2.04 | -2.26 | -2.73 | NM_004148 | Ninjurin 1 |
| 201438_at | COL6A3 | **down** | -2.04 | -2.34 | -2.93 | NM_004369 | Collagen, type VI, alpha 3 |
| 202478_at | TRIB2 | **down** | -2.04 | -2.42 | -5.2 | NM_021643 | Tribbles homolog 2 (Drosophila) |
| 218002_s_at | CXCL14 | **down** | -2.04 | -2.83 | -8.58 | NM_004887 | Chemokine (C-X-C motif) ligand 14 |
| 238933_at |  | **down** | -2.05 | -3.09 | -4.95 | AA644178 |  |
| 205406_s_at | SPA17 | **down** | -2.07 | -2.38 | -3.54 | NM_017425 | Sperm autoantigenic protein 17 |
| 217996_at | PHLDA1 | **down** | -2.07 | -2.51 | -2.98 | AA576961 | Pleckstrin homology-like domain, family A, member 1 |
| 206715_at | TFEC | **down** | -2.08 | -2.23 | -3.41 | NM_012252 | Transcription factor EC |
| 202994_s_at |  | **down** | -2.11 | -2.07 | -2.73 | Z95331 |  |
| 209099_x_at | JAG1 | **down** | -2.11 | -2.15 | -2.79 | U73936 | Jagged 1 (Alagille syndrome) |
| 216268_s_at | JAG1 | **down** | -2.11 | -2.22 | -2.78 | U77914 | Jagged 1 (Alagille syndrome) |
| 242873_at | KLRK1 | **down** | -2.11 | -2.99 | -5.44 | BE567130 | Killer cell lectin-like receptor subfamily K, member 1 |
| 209815_at | PTCH1 | **down** | -2.14 | -2.18 | -3.54 | BG054916 | Patched homolog 1 (Drosophila) |
| 203423_at | RBP1 | **down** | -2.14 | -2.47 | -3.62 | NM_002899 | Retinol binding protein 1, cellular |
| 207379_at | EDIL3 | **down** | -2.14 | -2.69 | -3.42 | NM_005711 | EGF-like repeats and discoidin I-like domains 3 |
| 212654_at | TPM2 | **down** | -2.15 | -2.04 | -2.4 | AL566786 | Tropomyosin 2 (beta) |
| 244353_s_at | SLC2A12 | **down** | -2.15 | -2.07 | -2.42 | AI675682 | Solute carrier family 2 (facilitated glucose transporter), memb 12 |
| 1568849_at | NLC1-B | **down** | -2.15 | -2.35 | -2.59 | BC009635 | Narcolepsy candidate region gene 1B |
| 202479_s_at | TRIB2 | **down** | -2.15 | -2.35 | -3.88 | BC002637 | Tribbles homolog 2 (Drosophila) |
| 203222_s_at | TLE1 | **down** | -2.15 | -2.47 | -3.61 | NM_005077 | Transducin-like enhancer of split 1 (E(sp1) homolog, Drosophila) |
| 231183_s_at |  | **down** | -2.16 | -2.22 | -2.27 | AI457817 | Transcribed locus |
| 212489_at | COL5A1 | **down** | -2.18 | -2.18 | -3.56 | AI983428 | Collagen, type V, alpha 1 |
| 202995_s_at | FBLN1 | **down** | -2.22 | -2.11 | -2.73 | NM_006486 | Fibulin 1 |
| 202755_s_at | GPC1 | **down** | -2.22 | -2.61 | -3.21 | AI354864 | Glypican 1 |
| 228367_at | ALPK2 | **down** | -2.22 | -3.37 | -5.47 | BE551416 | Alpha-kinase 2 |
| 206377_at | FOXF2 | **down** | -2.23 | -2.11 | -3.03 | NM_001452 | Forkhead box F2 |
| 223710_at | CCL26 | **down** | -2.26 | -3.16 | -7.75 | AF096296 | Chemokine (C-C motif) ligand 26 |
| 235210_s_at | RPESP | **down** | -2.27 | -2.6 | -3 | AW662373 | RPE-spondin |
| 1558803_at |  | **down** | -2.27 | -3.37 | -4.38 | BG253800 | CDNA FLJ41560 fis, clone CTONG1000088 |
| 222484_s_at | CXCL14 | **down** | -2.3 | -2.83 | -6.87 | AF144103 | Chemokine (C-X-C motif) ligand 14 |
| 242767_at | LMCD1 | **down** | -2.31 | -2.83 | -2.99 | N95437 | LIM and cysteine-rich domains 1 |
| 238604_at |  | **down** | -2.31 | -2.84 | -3.83 | AA768884 |  |
| 203919_at | TCEA2 | **down** | -2.33 | -2.23 | -2.84 | NM_003195 | Transcription elongation factor A (SII), 2 |
| 233413_at |  | **down** | -2.34 | -2.11 | -2.07 | AU156421 | CDNA FLJ13457 fis, clone PLACE1003343 |
| 210086_at | HR | **down** | -2.34 | -2.47 | -3 | AF039196 | Hairless homolog (mouse) |
| 217997_at | PHLDA1 | **down** | -2.34 | -2.77 | -3.81 | AI795908 | Pleckstrin homology-like domain, family A, member 1 |
| 224463_s_at | C11orf70 | **down** | -2.35 | -1.9 | -2.27 | BC006128 | Chromosome 11 open reading frame 70 |
| 203498_at | DSCR1L1 | **down** | -2.35 | -2.6 | -2.65 | NM_005822 | Down syndrome critical region gene 1-like 1 |
| 1560201_at | ZNF713 | **down** | -2.37 | -4.03 | -3.9 | AK097282 | Zinc finger protein 713 |
| 225842_at | PHLDA1 | **down** | -2.38 | -2.18 | -2.47 | AK026181 | Pleckstrin homology-like domain, family A, member 1 |
| 1555724_s_at | TAGLN | **down** | -2.38 | -2.93 | -3.87 | BC010946 | Transgelin |
| 209189_at | FOS | **down** | -2.38 | -3.14 | -2 | BC004490 | V-fos FBJ murine osteosarcoma viral oncogene homolog |
| 220922_s_at | SPANXA1 | **down** | -2.42 | -2.88 | -2.3 | NM_013453 | Sperm protein associated with the nucleus, X-linked, family member A1 |
| 209758_s_at | MFAP5 | **down** | -2.42 | -3.03 | -3.03 | U37283 | Microfibrillar associated protein 5 |
| 218000_s_at | PHLDA1 | **down** | -2.42 | -3.26 | -2.28 | NM_007350 | Pleckstrin homology-like domain, family A, member 1 |
| 1553027_a_at | KLHL4 | **down** | -2.42 | -3.37 | -3.57 | NM_057162 | Kelch-like 4 (Drosophila) |
| 228067_at | MGC42367 | **down** | -2.43 | -2.78 | -3.31 | AW249666 | Similar to 2010300C02Rik protein |
| 235763_at | SLC44A5 | **down** | -2.45 | -2.1 | -2.94 | AA001450 | Solute carrier family 44, member 5 |
| 220217_x_at | SPANXC | **down** | -2.46 | -2.6 | -2.34 | NM_022661 | SPANX family, member C |
| 204686_at | IRS1 | **down** | -2.46 | -3.31 | -5.86 | NM_005544 | Insulin receptor substrate 1 |
| 210886_x_at |  | **down** | -2.47 | -2.47 | -3.55 | AB007457 |  |
| 1557765_at | LOC643401 | **down** | -2.47 | -2.64 | -3.42 | BC039509 | Hypothetical protein LOC643401 |
| 244812_at |  | **down** | -2.47 | -2.84 | -4.31 | AA758116 | Transcribed locus, strongly similar to NP_444410.1 cycle related kinase [Mus musculus] |
| 1562926_at |  | **down** | -2.47 | -2.93 | -5.66 | BC033846 | Homo sapiens, clone IMAGE:4512650, mRNA |
| 213764_s_at | MFAP5 | **down** | -2.47 | -3.09 | -3.09 | AW665892 | Microfibrillar associated protein 5 |
| 217999_s_at | PHLDA1 | **down** | -2.5 | -2.48 | -2.41 | NM_007350 | Pleckstrin homology-like domain, family A, member 1 |
| 206363_at | MAF | **down** | -2.51 | -3.03 | -4.32 | NM_005360 | V-maf musculoaponeurotic fibrosarcoma oncogene homolog (avian) |
| 206985_at | HSD17B3 | **down** | -2.51 | -3.43 | -4.45 | NM_000197 | Hydroxysteroid (17-beta) dehydrogenase 3 |
| 206927_s_at | GUCY1A2 | **down** | -2.51 | -3.47 | -2.53 | NM_000855 | Guanylate cyclase 1, soluble, alpha 2 |
| 209351_at | KRT17 | **down** | -2.51 | -3.49 | -5.83 | BC002690 | Keratin 17 |
| 238127_at | FLJ41484 | **down** | -2.51 | -3.88 | -6.85 | AI479082 | Hypothetical protein LOC650669 |
| 209098_s_at | JAG1 | **down** | -2.52 | -2.01 | -3.15 | U61276 | Jagged 1 (Alagille syndrome) |
| 241337_at | LOC728347 | **down** | -2.54 | -2.18 | -9.35 | AI498602 | Hypothetical protein LOC728347 |
| 211965_at |  | **down** | -2.55 | -2.39 | -2.51 | BE620915 | Full-length cDNA clone CS0DI084YB21 of Placenta Cot 25-normalized of Homo sapiens (human) |
| 235209_at | RPESP | **down** | -2.55 | -2.54 | -3.02 | AW662373 | RPE-spondin |
| 213765_at | MFAP5 | **down** | -2.55 | -2.88 | -2.98 | AW665892 | Microfibrillar associated protein 5 |
| 235392_at | IRS1 | **down** | -2.55 | -3.75 | -6.51 | BG403162 | Insulin receptor substrate 1 |
| 1560527_at |  | **down** | -2.57 | -2.34 | -2.65 | BU587810 |  |
| 205547_s_at | TAGLN | **down** | -2.59 | -2.98 | -3.8 | NM_003186 | Transgelin |
| 201340_s_at | ENC1 | **down** | -2.64 | -3.09 | -2.42 | AF010314 | Ectodermal-neural cortex (with BTB-like domain) |
| 1568765_at | SERPINE1 | **down** | -2.65 | -2.36 | -2.18 | BC020765 | Serpin peptidase inhibitor, clade E (nexin, plasminogen activator inhibitor type 1 |
| 1562909_at | C1orf98 | **down** | -2.67 | -2.21 | -3.79 | BC040731 | Chromosome 1 open reading frame 98 |
| 219302_s_at | CNTNAP2 | **down** | -2.68 | -2.15 | -4.08 | NM_014141 | Contactin associated protein-like 2 |
| 210241_s_at |  | **down** | -2.69 | -2.64 | -4.25 | AB007458 |  |
| 209917_s_at | TP53AP1 | **down** | -2.69 | -3.31 | -4.94 | BC002709 | TP53 activated protein 1 |
| 242979_at |  | **down** | -2.74 | -2.83 | -6.88 | AI474666 |  |
| 236335_at |  | **down** | -2.8 | -3.32 | -5.39 | AW298375 | CDNA clone IMAGE:5273964 |
| 224032_x_at | SPANXA1 | **down** | -2.88 | -3.37 | -2.78 | AF098306 | Sperm protein associated with the nucleus, X-linked, family member A1 |
| 228731_at |  | **down** | -2.89 | -2.39 | -5.07 | AW236803 | CDNA clone IMAGE:5273964 |
| 235889_at |  | **down** | -2.9 | -2.16 | -2.69 | AI825987 | Transcribed locus |
| 228038_at | SOX2 | **down** | -2.98 | -2.69 | -4.86 | AI669815 | SRY (sex determining region Y)-box 2 |
| 227134_at | SYTL1 | **down** | -2.98 | -2.98 | -5.2 | AI341537 | Synaptotagmin-like 1 |
| 203504_s_at | ABCA1 | **down** | -2.99 | -2.38 | -2.26 | NM_005502 | ATP-binding cassette, sub-family A (ABC1), member 1 |
| 1555673_at |  | **down** | -3.03 | -4.85 | -2.93 | BC012486 |  |
| 224724_at | SULF2 | **down** | -3.05 | -2.61 | -3.67 | AL133001 | Sulfatase 2 |
| 1570469_at |  | **down** | -3.13 | -3.5 | -5.1 | BC017988 | Homo sapiens, clone IMAGE:4246712, mRNA |
| 218211_s_at | MLPH | **down** | -3.24 | -2.49 | -3.19 | NM_024101 | Melanophilin |
| 214366_s_at | ALOX5 | **down** | -3.31 | -3.56 | -6.91 | AA995910 | Arachidonate 5-lipoxygenase |
| 209348_s_at | MAF | **down** | -3.31 | -4.36 | -5.49 | AF055376 | V-maf musculoaponeurotic fibrosarcoma oncogene homolog (avian) |
| 229327_s_at | MAF | **down** | -3.32 | -3.97 | -3.43 | BE674528 | V-maf musculoaponeurotic fibrosarcoma oncogene homolog (avian) |
| 229831_at | CNTN3 | **down** | -3.4 | -4.57 | -2.61 | BE221817 | Contactin 3 (plasmacytoma associated) |
| 227449_at | EPHA4 | **down** | -3.44 | -4.16 | -4.38 | AI799018 | EPH receptor A4 |
| 229810_at |  | **down** | -3.5 | -2.47 | -2.35 | AI796536 | Transcribed locus |
| 206114_at | EPHA4 | **down** | -3.54 | -3.69 | -2.11 | NM_004438 | EPH receptor A4 |
| 204446_s_at | ALOX5 | **down** | -3.74 | -4.44 | -8.17 | NM_000698 | Arachidonate 5-lipoxygenase |
| 212636_at |  | **down** | -3.88 | -4.02 | -3.42 | AL031781 |  |
| 214079_at | DHRS2 | **down** | -4.14 | -6.97 | -10.56 | AK000345 | Dehydrogenase/reductase (SDR family) member 2 |
| 1566147_a_at |  | **down** | -4.23 | -3.88 | -4.29 | AK098337 | CDNA FLJ41018 fis, clone UTERU2018881 |
| 237094_at | FAM19A5 | **down** | -4.27 | -3.09 | -2.27 | AI953086 | Family with sequence similarity 19 (chemokine (C-C motif)-like), member A5 |
| 228186_s_at | RSPO3 | **down** | -4.37 | -5.5 | -14.14 | BF589322 | R-spondin 3 homolog (Xenopus laevis) |
| 214761_at | ZNF423 | **down** | -4.7 | -2.45 | -2.85 | AW149417 | Zinc finger protein 423 |
| 205919_at | HBG2 | **down** | -4.71 | -3.94 | -2.51 | NM_005330 | Hemoglobin, gamma G |
| 213415_at | CLIC2 | **down** | -5.36 | -3.39 | -2.49 | AI768628 | Chloride intracellular channel 2 |
| 229655_at | FAM19A5 | **down** | -5.87 | -4.12 | -3.5 | N66656 | Family with sequence similarity 19 (chemokine (C-C motif)-like), member A5 |
| 203074_at | ANXA8 | **down** | -6.18 | -7.01 | -8.99 | NM_001630 | Annexin A8 |
| 221690_s_at | NLRP2 | **down** | -6.25 | -12.99 | -10.9 | AF298547 | NLR family, pyrin domain containing 2 |
| 229459_at | FAM19A5 | **down** | -6.3 | -4 | -3.04 | AV723914 | Family with sequence similarity 19 (chemokine (C-C motif)-like), member A5 |
| 207174_at | GPC5 | **down** | -6.86 | -14.9 | -4.73 | NM_004466 | Glypican 5 |
| 1556395_at |  | **down** | -7.21 | -5.79 | -5.66 | AF147356 | Full length insert cDNA clone YB44H10 |
| 206463_s_at | DHRS2 | **down** | -7.21 | -13.71 | -18.38 | NM_005794 | Dehydrogenase/reductase (SDR family) member 2 |
| 244276_at | KLB | **down** | -7.58 | -13.61 | -11.26 | AI668605 | Klotho beta |
| 205268_s_at | ADD2 | **down** | -7.86 | -6.57 | -11.33 | NM_017488 | Adducin 2 (beta) |
| 225062_at | FLJ36840 | **down** | -11.92 | -8.27 | -11.41 | BF667120 | Hypothetical LOC645524 |
| 210546_x_at | CTAG1B | **down** | -13.01 | -7.93 | -12.69 | U87459 | Cancer/testis antigen 1B |
| 225046_at |  | **down** | -13.33 | -12.39 | -10.72 | AL521247 | Transcribed locus |
| 223235_s_at | SMOC2 | **down** | -14.6 | -12.62 | -10.6 | AB014737 | SPARC related modular calcium binding 2 |
| 215733_x_at | CTAG2 | **down** | -15.89 | -16.46 | -13.45 | AJ012833 | Cancer/testis antigen 2 |
| 1554072_s_at | CCDC67 | **down** | -20.24 | -5.87 | -35.21 | BC031247 | Coiled-coil domain containing 67 |
| 206421_s_at | SERPINB7 | **down** | -23.05 | -25.43 | -32.1 | NM_003784 | Serpin peptidase inhibitor, clade B (ovalbumin), member 7 |
| 204664_at | ALPP | **down** | -26.07 | -28.73 | -41.82 | NM_001632 | Alkaline phosphatase, placental (Regan isozyme) |
| 228598_at | DPP10 | **down** | -38.64 | -4.16 | -2.51 | AL538781 | Dipeptidyl-peptidase 10 |
| 236313_at | CDKN2B | **down** | -40.27 | -9.96 | -37.19 | AW444761 | Cyclin-dependent kinase inhibitor 2B (p15, inhibits CDK4) |
| 227711_at | FAM112B | **down** | -63.7 | -26.78 | -18.95 | BG150433 | Family with sequence similarity 112, member B |
| 235557_at | LOC150763 | **down** | -69.91 | -80.7 | -65.39 | AW082827 | Hypothetical protein LOC150763 |
