# Supplementary material for: The Tumor Suppressor PRDM5 Regulates Wnt Signaling at Early Stages of Zebrafish Development
Source: PLoS One. 2009 Jan 26;4(1):e4273. doi: 10.1371/journal.pone.0004273 (PMC2627919; doi:10.1371/journal.pone.0004273)
Supplement: Table S2 — Validation of microarray results by qPCR. GeneChip predictions are shown in the first three columns. Fold changes (FC) predicted by Affymetrix and by qPCR are shown. (0.09 MB DOC) [file pone.0004273.s004.doc]

**Table S2.** Validation of microarray results by qPCR. GeneChip predictions are shown in the first three columns. Fold changes (FC) predicted by Affymetrix and by qPCR are shown.

| **Validation of GeneChip results in U2OS cells** | | | | | | | | | | | |
| --- | --- | --- | --- | --- | --- | --- | --- | --- | --- | --- | --- |
|  | **GeneChip results** | | |  | **FC** | | |  | **TaqMan results** | | |
|  | | | | | | | | | | | |
| **Gene** | **8** | **24** | **48** |  | **8** | **24** | **48** |  | **8** | **24** | **48** |
|  |  |  |  |  |  |  |  |  |  |  |  |
| **DKK1** | **up** | **up** | **up** |  | 2,3 | 2,7 | 7,2 |  | -1,2 | 2,2 | 1,9 |
| **EPAS1** |  |  | **up** |  | 0,0 | 1,7 | 3,3 |  | 1,7 | 6,9 | 5,1 |
| **HDAC9** |  |  | **up** |  | 1,3 | 1,3 | 2,4 |  | 1,6 | 3,5 | 3,3 |
| **IL1A** |  | **up** | **up** |  | 3,3 | 12,6 | 10,3 |  | 6,0 | 17,5 | 36,6 |
| **IL24** | **up** |  | **up** |  | 2,9 | 1,4 | 2,4 |  | 5,0 | 4,0 | 4,1 |
| **IL8** | **up** | **up** | **up** |  | 2,9 | 2,7 | 13,3 |  | 4,2 | 6,7 | 36,7 |
| **KREMEN1** |  | **up** | **up** |  | -0,4 | 1,9 | 2,5 |  | -1,1 | 2,7 | 3,2 |
| **TNFRSF10B** |  |  | **up** |  | -0,9 | 1,1 | 1,9 |  | 1,4 | 2,4 | 2,4 |
|  |  |  |  |  |  |  |  |  |  |  |  |
| **CAV1** | **down** | **down** | **down** |  | -2,0 | -2,6 | -3,4 |  | -2,1 | -2,1 | -4,9 |
| **CCND1** |  |  | **down** |  | -1,5 | -1,9 | -2,1 |  | -1,1 | -0,9 | -2,2 |
| **DDIT4** |  |  | **down** |  | -1,3 | -1,9 | -2,8 |  | -1,2 | -0,8 | -2,5 |
| **ENC1** | **down** | **down** | **down** |  | -2,3 | -2,6 | -2,5 |  | -1,8 | -2,9 | -5,1 |
| **FZD1** |  |  | **down** |  | -1,6 | -1,8 | -2,7 |  | 1,1 | -1,8 | -2,3 |
| **GPC1** | **down** | **down** | **down** |  | -2,0 | -2,5 | -3,4 |  | -1,7 | -1,8 | -3,1 |
| **HES1** |  | **down** | **down** |  | -1,7 | -2,3 | -3,3 |  | -1,6 | -1,3 | -2,5 |
| **HES6** | **down** | **down** |  |  | -12,8 | -18,6 | -17,0 |  | -24,0 | -7,0 | -11,9 |
| **JAG1** | **down** | **down** | **down** |  | -2,3 | -2,1 | -2,8 |  | -2,1 | -1,3 | -4,6 |
| **JUN** |  | **down** | **down** |  | -1,6 | -2,3 | -2,1 |  | -2,3 | -1,4 | -2,4 |
| **RAD52** |  |  | **down** |  | -1,5 | -1,4 | -2,7 |  | -1,2 | -0,8 | -2,1 |
| **ROR1** |  |  | **down** |  | -1,5 | -1,9 | -2,8 |  | -1,9 | -1,7 | -3,3 |
| **RSPO3** | **down** | **down** | **down** |  | -4,4 | -5,5 | -14,1 |  | -3,4 | -7,7 | -20,0 |
| **SERPINB5** |  | **down** | **down** |  | -9,7 | -11,0 | -6,4 |  | -30,7 | -13,7 | -8,4 |
| **SOX2** | **down** | **down** | **down** |  | -2,7 | -2,7 | -3,9 |  | -2,4 | -4,9 | -7,5 |
| **SOX4** |  | **down** | **down** |  | -1,4 | -2,3 | -4,0 |  | -1,5 | -1,6 | -7,1 |
| **TCF3** |  |  | **down** |  | -1,3 | -1,5 | -2,2 |  | -1,1 | -1,0 | -0,9 |
| **TGFB2** |  | **down** | **down** |  | -1,4 | -2,3 | -3,5 |  | -1,3 | -1,6 | -3,7 |
| **TLE1** | **down** | **down** | **down** |  | -1,9 | -2,3 | -3,0 |  | -1,7 | -0,9 | -2,4 |
| **TOX** |  | **down** | **down** |  | -1,8 | -2,4 | -2,5 |  | -2,0 | -1,2 | -2,6 |
