# Supplementary material for: The Tumor Suppressor PRDM5 Regulates Wnt Signaling at Early Stages of Zebrafish Development
Source: PLoS One. 2009 Jan 26;4(1):e4273. doi: 10.1371/journal.pone.0004273 (PMC2627919; doi:10.1371/journal.pone.0004273)
Supplement: Table S3 — Functional classification of genes regulated by PRDM5 in U2OS cells. (0.16 MB DOC) [file pone.0004273.s005.doc]

**Table S3.** Functional classification of genes regulated by PRDM5 in U2OS cells.

| **Symbol** | **Cellular**  **component** | **Function** | **Biological process** | **Expression** |
| --- | --- | --- | --- | --- |
| **Decreased Expression** | | | | |
| ABCA1 | membrane | cholesteral efflux pump in the cellular lipid removal pathway. Mutations associated with Tangier's disease and familial high-density lipoprotein deficiency |  | widely expressed, but most abundant in macrophages |
| ADD2 | membrane | membrane skeletal proteins assembly of the spectrin-actin network. binds to calmodulin |  | NS and hematopoietic tissues |
| ALOX5 | cytoplasm | inflammatory response,synthesis of leukotrienes |  | bone marrow-derived cells |
| ALPK2 |  | protein serine/threonine kinase activity |  |  |
| ALPP | membrane | alkaline phosphatase activity |  | placenta |
| ANXA8 |  | Ca (2+) dependent phospholipid binding protein |  | lung endothelia, skin, liver, and kidney;overexpressed in AML |
| CAV1 | membrane | scaffolding protein of unknown function; membrane protein of caveolae |  |  |
| CCDC67 | unknown | integral component of the filamentous matrix of the centrosome |  |  |
| CCL26 | secreted protein | chemotactic for eosinophils and basophils. binds to ccr3 | chemotactic activity |  |
| CDKN2B | cytoplasm | negative regulation cell cycle progression | cell cycle |  |
| CLIC2 | membrane | ion transport | cell homeostasis | fetal liver and adult skeletal muscle tissue |
| CNTN3 | membrane | can mediate cell surface interactions during nervous system development | cell adhesion | NS |
| CNTNAP2 | membrane | cell adhesion;neurexin family of receptor | may play a role in the local differentiation of the axon into distinct functional subdomains | NS/embryonic tissue |
| COL5A1 | extracellular matrix | binds to dna, heparan sulfate, thrombospondin, heparin, and insulin. | cell adhesion | ubiquitous |
| COL6A3 | extracellular matrix | cell adhesion | cell adhesion and development | embryonic tissue |
| CTAG1B | membrane |  |  | tumor specific antigen |
| CTAG2 | membrane |  |  | tumor specific antigen |
| CXCL14 | secreted protein | chemotaxis |  |  |
| DHRS2 | nucleus | metabolism; may negatively regulate cell cycle progression |  |  |
| DPP10 | membrane | serine protease with no detectable protease activity |  |  |
| DSCR1L1 | unknown | signal trasduction | development | NS |
| EBF1 | nucleus | transcription | development |  |
| EDIL3 | secreted protein | integrin ligand;angiogenesis/vessel dev | cell adhesion | NS |
| ENC1 | nucleus | p53 induced gene and WNT induced gene | development | NS |
| EPHA4 | membrane | TK receptor subfamily; signaling activity | development | NS |
| FBLN1 | extracellular matrix | ECM remodelling | cell adhesion |  |
| FOXF2 | nucleus | regulation of wnt signaling and promoting extracellular matrix production | development | gut |
| GABRB3 | membrane | receptor for inhibitory neurotransmitter; subunits of chloride channel | neuronal inhibition | NS |
| GAS1 | membrane | negative regulation cell cycle progression (blocks entry in S) | cell cycle |  |
| GPC1 | membrane | signaling activity | development |  |
| GPC5 | membrane | signaling activity | development | NS |
| GUCY1A2 | cytoplasm | signal trasduction; guanylyl cyclase |  |  |
| HBG2 | cytoplasm | oxygen transport | cell homeostasis | red blood cells |
| HR | nucleus | transcription factor | growth |  |
| HSD17B3 |  | steroid biosynthesis | development | testis |
| IRS1 | cytoplasm | signal trasduction |  |  |
| JAG1 | membrane | Notch ligand | development and growth |  |
| KLB | cytoplasm | regulation of calcium and phosphorus homeostasis and FGF signalling | vitamin D metabolism | kidney |
| KLHL4 |  | putative actin-binding |  |  |
| KLRK1 | membrane | involved in the immune surveillance exerted by T-and B-lymphocytes. |  | NK cells |
| KRT17 | cytoplasm | intermediate filament | hair and nail development |  |
| LMCD1 | nucleus | transcription; transcription co-factor | transcriptional cofactor that restricts GATA6 function by inhibiting DNA binding | lung and cardiac tissue |
| MAF | nucleus | transcription factor-protooncogene (MM; lymphoma) | lens development |  |
| MFAP5 | extracellular matrix | structural components of ECM | mediate interactions between fibrillin-containing microfibrils and cell surfaces; notch signaling activation |  |
| MGC42367 |  |  |  |  |
| MLPH | cytoplasm | melanosome transport | Rab GTPase and myosin Va binding |  |
| NINJ1 | membrane | axonal growth; senescence induction; protein binding | cell adhesion and development | NS |
| NLC1-B |  |  |  |  |
| NLRP2 |  | modulator of the activation of NF-kappaB and pro-caspase-1 in macrophages | inflammation; DNA damage response |  |
| PHLDA1 |  | mediator of anti-apoptotic effects of insulin-like growth factor I |  |  |
| PTCH1 | membrane | receprtor for SHH signalling | development and cell cycle |  |
| QKI |  | rna export from the nucleus; RNA-binding protein essential for myelination of the central nervous system | oligodendrocyte lineage development | NS |
| RBP1 | cytoplasm | intracellular transport of retinol; carrier | vitamin A metabolism |  |
| RHOBTB3 | cytoplasm | RHOBTB subfamily of Rho GTPases |  |  |
| RPESP |  | PRE-spondin |  |  |
| RSPO3 | extracellular matrix | promotion of Wnt/beta-catenin signaling pathway | development | NS |
| SERPINB7 | secreted protein | endopeptidase inhibitor |  |  |
| SERPINE1 | secreted protein | endopeptidase inhibitor |  |  |
| SLC2A12 | membrane/cytoplasm | glucose transporter |  | kidney |
| SLC44A5 |  | transporter |  |  |
| SMOC2 | extracellular matrix | matricellular protein, may regulate interactions between cells and the extracellular matrix | induces mitogenesis and angiogenesis of endothelial cells |  |
| SOX2 | nucleus | transcription; sox2 mutations cause anophthalmia (absence of one or both eyes) | development | NS |
| SPA17 | membrane | promotes fertilization | cell adhesion | cancer antigen |
| SPANXA1 | nucleus |  | spermatozoa maturation |  |
| SPANXC | nucleus |  | spermatozoa maturation |  |
| SULF2 | extracellular matrix | glycan editing: signalling |  |  |
| SYTL1 | cytoplasm | cell signalling, secretory pathways and membrane dynamics; adaptor protein |  |  |
| TAGLN | cytoplasm | actin cross-linking protein involved in change in cell shape |  |  |
| TCEA2 | nucleus | responsible for releasing RNA polymerase II ternary complexes from transcriptional arrest at template-encoded arresting sites | transcriptional elongation |  |
| TCFL5 | nucleus | transcription factor | development |  |
| TFEC | nucleus | transcription factor | development | myeloid compartment |
| TLE1 | nucleus | negative reg WNT signaling; transcription co-factor | development |  |
| TP53AP1 | nucleus | p53 induced gene |  |  |
| TPM2 | cytoplasm | actin binding |  |  |
| TRIB2 | cytoplasm | regulates MAPK kinase activation | oncogenic role in AML |  |
| ZNF423 | nucleus | transcription factor | cerebellar development | NS |
| ZNF713 |  |  |  |  |
| **Increased Expression** | | | | |
| ADAMTS6 | extracellular matrix | ECM remodelling; peptidase activity | development |  |
| B3GNT5 | membrane | glycolipid biosynthesis; transferase activity | metabolism |  |
| CCL20 | secreted protein | negative regulation of cell proliferation; chemochine activity | chemotaxis |  |
| CD33 | membrane | cell adhesion; negative regulation of cell proliferation; receptor acivity | cell proliferaion |  |
| DKK1 | secreted protein | inhibition of WNT signaling | development |  |
| ERBB3 | membrane | receptor protein wo TK activity for neuregulins; receptor acivity | growth & differentiation |  |
| ETV1 | nucleus | neuronal identity; transcription factor | development | NS |
| HCLS1 | membrane | antigen receptor signaling |  |  |
| HIST1H2BG | nucleus | nucleosome component | nucleosome assembly |  |
| HIST1H4H | nucleus | nucleosome component | nucleosome assembly |  |
| HIST2H2AA3 | nucleus | nucleosome component | nucleosome assembly |  |
| IL1RAPL1 | membrane | interleukin 1 receptor family | development |  |
| IL32 | secreted protein | inducer of tnf-alpha and il8; ligand activity | chemotaxis |  |
| IL8 | secreted protein | angiogenesis and proliferation; ligand activity | chemotaxis |  |
| INSL4 | secreted protein | placental apoptosis; ligand activity | development |  |
| KISS1 | secreted protein | inhibitor of cell motility;suppressor of tumour metastasis; ligand activity | growth & metastasis |  |
| LAMA1 | extracellular matrix | costituent of basament membrane | lens development | ocular structures, lens |
| MEGF10 | membrane | phagocytosis of dead cells; receptor activity |  |  |
| OSR2 | nucleus | transcriptional regulation of kidney development | development |  |
| PAX8 | nucleus | transcriptional regulation of kidney development | development |  |
| PRSS1 | secreted protein | peptidase activity |  |  |
| PRTFDC1 |  |  |  |  |
| PTPN7 | cytoplasm | TCR signaling; phosphatase activity |  |  |
| PTPRR | cytoplasm | modulation of signal trasduction; phosphatase activity | neuronal growth and differentiation |  |
| SCG5 | secreted protein | neuropeptide signaling pathway |  |  |
| SERPINF1 | secreted protein | positive regulator of neurogenesis, differentiation & apoptosis; negative reg of angiogenesis; inhibitor of peptidase activity | development |  |
| SLAMF7 | membrane | cell adhesion | cell adhesion/NK cell activation |  |
| SLC26A6 | membrane | transporter |  |  |
| STC1 | secreted protein | regulation of cellular calcium/phosphate homeostasis | metabolism |  |
| TCEAL7 | nucleus | inducer of apoptosis; transcription elongation factor |  |  |
| THOC1 | nucleus | RNA processing and export from the nucleus |  |  |
| VSTM1 |  |  |  |  |
