# Supplementary material for: The Tumor Suppressor PRDM5 Regulates Wnt Signaling at Early Stages of Zebrafish Development
Source: PLoS One. 2009 Jan 26;4(1):e4273. doi: 10.1371/journal.pone.0004273 (PMC2627919; doi:10.1371/journal.pone.0004273)
Supplement: Table S4 — PRDM5 target genes of the wnt pathway. (0.08 MB DOC) [file pone.0004273.s006.doc]

**Table S4.** PRDM5 target genes of the wnt pathway.

| **Gene Symbol** | **wnt**  **signalling** | **wnt targets** | **wnt antagonists** | **wnt agonists** | **PCP signalling** | **Related signalling** | **8 hr** | **24 hr** | **48 hr** |
| --- | --- | --- | --- | --- | --- | --- | --- | --- | --- |
| **CDH2** | **+** |  |  |  |  |  |  |  | **down** |
| **FZD1** | **+** |  |  |  |  |  |  |  | **down** |
| **TCF4** | **+** |  |  |  |  |  |  |  | **down** |
| **TLE1** | **+** |  |  |  |  |  | **down** | **down** | **down** |
| **KREMEN1** | **+** |  | **+** |  |  |  |  | **up** | **up** |
| **WNT4** | **+** |  |  |  |  |  |  | **up** |  |
| **CCND1** |  | **+** |  |  |  |  |  |  | **down** |
| **ENC1** |  | **+** |  |  |  |  | **down** | **down** | **down** |
| **FGF9** |  | **+** |  |  |  |  |  |  | **down** |
| **FN1** |  | **+** |  |  |  |  |  | **down** | **down** |
| **FST** |  | **+** |  |  |  |  |  | **down** | **down** |
| **GJA1** |  | **+** |  |  |  |  |  |  | **down** |
| **JAG1** |  | **+** |  |  |  |  | **down** | **down** | **down** |
| **JUN** |  | **+** |  |  |  |  |  | **down** | **down** |
| **L1CAM** |  | **+** |  |  |  |  |  |  | **down** |
| **SOX11** |  | **+** |  |  |  |  |  | **down** | **down** |
| **SOX2** |  | **+** | **+** |  |  |  | **down** | **down** | **down** |
| **IL8** |  | **+** |  |  |  |  | **up** | **up** | **up** |
| **DACT1** |  |  | **+** |  |  |  |  |  | **down** |
| **SOX9** |  |  | **+** |  |  |  |  |  | **down** |
| **DKK1** |  | **+** | **+** |  |  |  | **up** | **up** | **up** |
| **DKK2** |  |  | **+** |  |  |  |  | **up** |  |
| **RSPO3** |  |  |  | **+** |  |  | **down** | **down** | **down** |
| **SOX4** |  |  |  | **+** |  |  |  | **down** | **down** |
| **DAAM2** |  |  |  |  | **+** |  |  |  | **down** |
| **GPC1** |  |  |  |  | **?** |  | **down** | **down** | **down** |
| **GPC5** |  |  |  |  | **?** |  | **down** | **down** | **down** |
| **ROR1** |  |  |  |  | **+** |  |  |  | **down** |
| **WNT5A** |  |  |  |  | **+** |  | **up** |  |  |
| **ACVR1B** |  |  |  |  |  | **+** |  |  | **up** |
| **ACVR1C** |  |  |  |  |  | **+** |  |  | **up** |
| **TGFB2** |  |  |  |  |  | **+** |  | **down** | **down** |
