# Supplementary material for: The Tumor Suppressor PRDM5 Regulates Wnt Signaling at Early Stages of Zebrafish Development
Source: PLoS One. 2009 Jan 26;4(1):e4273. doi: 10.1371/journal.pone.0004273 (PMC2627919; doi:10.1371/journal.pone.0004273)
Supplement: Table S5 — prdm5 depletion enhances masterblind phenotype in axin+/− zebrafish embryos. Three independent experiments of rescue of the mbl phenotype are shown. Control: not injected embryos; Mix mo: embryos injected with ATG and SB mo; mRNA: embryos injected with hPRDM5 mRNA. The expected percentage of normal or mbl embryos is shown (EXPECTED); the number (and percentage) of embryos obtained in each experiment and the corresponding phenotype is reported (EXP1, EXP2, EXP3, PHENOTYPE). (0.04 MB DOC) [file pone.0004273.s007.doc]

**Table S5. *prdm5* depletion enhances *masterblind* phenotype in *axin*+/- zebrafish embryos.** Three independent experiments of rescue of the *mbl* phenotype are shown. Control: not injected embryos; Mix mo: embryos injected with ATG and SB mo; mRNA: embryos injected with *hPRDM5* mRNA. The expected percentage of normal or *mbl* embryos is shown (EXPECTED); the number (and percentage) of embryos obtained in each experiment and the corresponding phenotype is reported (EXP1, EXP2, EXP3, PHENOTYPE).

| **SAMPLES** | **EXPECTED** | **EXP. 1** | **EXP. 2** | **EXP. 3** | **PHENOTYPE** |
| --- | --- | --- | --- | --- | --- |
|  |  |  |  |  |  |
| **control** | **75%** | **7 (78%)** | **24 (73%)** | **13 (87%)** | **normal** |
|  | **25%** | **2 (22%)** | **9 (27%)** | **2 (13%)** | **mbl** |
|  |  | **9** | **33** | **15** |  |
|  |  |  |  |  |  |
| **Mix mo** | **75%** | **6 (15%)** | **20 (26%)** | **21(62%)** | **normal** |
|  |  | **14 (34%)** | **21 (27%)** | | **very small eyes** |
|  | **25%** | **21 (51%)** | **36 (47%)** | **13 (38%)** | **mbl** |
|  |  | **41** | **77** | **34** |  |
|  |  |  |  |  |  |
| **mRNA** | **75%** | **92 (72%)** | **40 (73%)** | **36 (68%)** | **normal** |
|  | **25%** | **35 (28%)** | **15 (27%)** | **17 (32%)** | **mbl** |
|  |  | **127** | **55** | **53** |  |
|  |  | **46 (36%)** | **25 (45%)** | **24 (45%)** | **Of all mRNA injected embryos: short body & big head** |
